# Corrosion Crack Morphology and Creep Analysis of Members Based on Meso-Scale Corrosion Penetration

**DOI:** 10.3390/ma15207338

**Published:** 2022-10-20

**Authors:** Bin Zeng, Yiping Yang, Fuyuan Gong, Koichi Maekawa

**Affiliations:** 1 Central Research Institute of Building and Construction Co., Ltd., MCC Group, Beijing 100088, China; 2 College of Civil Engineering and Architecture, Zhejiang University, Hangzhou 310058, China; 3 Institute of Urban Innovation, Yokohama National University, Yokohama 240-8501, Japan

**Keywords:** corrosion cracking mode, corrosion product penetration, creep behavior, plastic damage deformation, rate-dependent constitutive model, reinforced concrete beam

## Abstract

In this paper, to study the development of load-carrying capacity and long-term creep performance of reinforced concrete beams under different corrosion patterns, the rate-dependent model of concrete is used as the basis to consider the creep development process from the meso-scale level. The porosity mechanics method is used to simulate the generation and penetration process of corrosion products. Three corrosion conditions are set: bottom longitudinal reinforcement corrosion, top longitudinal reinforcement corrosion and all reinforcement corrosion. The corrosion rate is used as the variable in each corrosion condition. The results show that: (1) the greater the corrosion rate in all conditions, the lower the bearing capacity. In addition, the corrosion of top longitudinal reinforcement causes the damage form of the beam to change to brittle damage; (2) the creep coefficient decreases with the increase in corrosion rate in all working conditions, but the main factor for this phenomenon is the obvious increase in initial deformation. Consequently, it is not suitable to follow the conventional creep concept (deformation development/initial deformation) for the development of plastic deformation of damaged members. It is more reasonable to use the global deflection to describe the long-term deformation of corrosion-damaged members.

## 1. Introduction

Reinforced concrete structures are widely used in houses, tunnels, ports, and other infrastructure construction. These RC structures located in the coastal area are in a complex environment coupled with corrosion influence and load effect. Rust of reinforcement bars in concrete causes internal micro-cracks and cumulative damage. Corrosion not only reduces the elastic modulus and ultimate bearing capacity of structure members but also makes the structure deformation increase and then affects the long-term creep performance of the structure.

There are few research results about the influence of rust cracking on the creep development of reinforced concrete members. Cao [1] conducted several groups on axial compression creep experiments of corroded concrete columns with different corrosion rates and found that the greater the corrosion rate of reinforcement, the greater the creep deformation of components, which was attributed to the weakening of the constraint effect of corroded reinforcement on concrete. Yoon et al. [2] carried out the sustained load test of a steel concrete beam soaked in salt solution after electrical-corrosion treatment. The results show that the deflection of the energized specimens increases in a stepwise manner compared with that of the non-energized specimens, and the corrosion and creep coupling failure occur when the loading level is high. Shen et al. [3] studied the damage development of reinforced concrete beams under the simultaneous action of electrical corrosion and sustained load. The results show that corrosion will change the surface crack distribution and reduce the residual bearing capacity of beam members. At present, the systematic research on the influence of rust on creep at the component level is not sufficient, and the relevant research usually only focuses on the change in rust rate, and the influence of different rust cracking modes on creep needs to be studied urgently.

Through the self-developed multi-scale model of material structure, the coupling analysis of the hydration process, material transport, structural durability deterioration and mechanical property deterioration of concrete materials is realized [4,5]. The calculation of the corrosion phenomenon of reinforced concrete structures can simulate the formation and penetration process of corrosion products and the development process of rust expansion cracks in reinforcement concrete, which reflects the influence of rust expansion cracks on the mechanical constitutive model of concrete and reflects the performance degradation mechanism of corroded members. Thus, structural responses such as the rust expansion phenomenon and mechanical property degradation of rusted reinforced concrete beams under rusted conditions can be reasonably explained [5,6,7], which makes up for the defect that the existing corrosion simulation studies do not consider the penetration of rust products [8,9]. The creep calculation is based on the rate creep constitutive model of meso-mechanics [10,11,12], which deals with the influence of pore moisture change on concrete creep from the meso-level [13,14] and can reflect the creep development process of actual concrete members.

As mentioned before, research on the creep development of rust-damaged members is relatively limited, and the only studies available usually only consider the effect of the corrosion rate on the mechanical properties of the beams. In this paper, based on the concrete material rate-dependent model considering the time course effect of creep, coupled with the analysis of corrosion product entity generation and infiltration process, the changes of load-bearing capacity and creep long-term performance of reinforced concrete beams under different rust expansion and cracking modes are investigated. The influence law of rust rate and rust morphological characteristics on the creep properties of steel and concrete structures is explored in depth.

## 2. Calculation of Creep and Corrosion Cracking

### 2.1. Creep Constitutive Model

**Figure 1:**
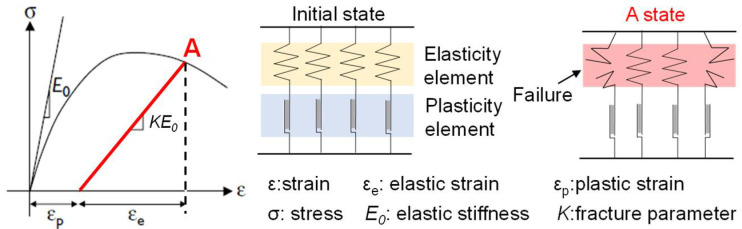
Mechanical model for elastic-plastic damage of concrete [4].

Based on the author’s previous research, the rate-type elastic-plastic damage constitutive model of concrete material is shown in Figure 1 [4]. The mechanical unit of concrete material is regarded as a parallel model of multiple Maxwell elements, and the total stress is the sum of stresses of all elements, and the total strain is equal to the strain of each element. The nonlinear deformation characteristics of concrete are characterized by damage coefficient *K* and plastic strain *ε_p_*. *K* is the ratio of the number of remaining intact elements after damage to the number of initial elements, and *ε_p_* is the plastic deformation of the plastic parts of each Maxwell element. The damage coefficient *K* and plastic strain *ε_p_* represent the damage degree and plastic deformation of the material, respectively, which determine the change in elastic modulus and plastic strain of concrete materials.

The basic mathematical expression of the elastic-plastic damage mechanical model of concrete is shown in Equation (1).

(1) $$\varepsilon =\varepsilon_{e}+\varepsilon_{p},\;\sigma =E_{0}\varepsilon_{e}K$$

The influence of the damage coefficient and plastic strain is expressed by the rate-type formula, see Equation (2), where the first term is the time history change in plastic deformation (i.e., creep), and the second term is the cumulative deformation and damage of cyclic action (i.e., fatigue). The creep constitutive of concrete is proposed based on the above elastoplastic damage mechanical model of concrete. The calculation formula of creep can be obtained by considering the linear creep of concrete into the creep constitutive model, as shown in Equation (3). The first term is the time history law of plastic deformation of concrete material, which reflects the nonlinear creep of concrete material. *Φ* represents the tendency of plastic deformation to increase and decrease in the development of life history, while the second term *κ* is linear creep.

(2) $$d\varepsilon_{p}=\left(\frac{\partial \varepsilon_{p}}{\partial t}\right)dt+\left(\frac{\partial \varepsilon_{p}}{\partial \varepsilon_{e}}\right)d\varepsilon_{e},dK=\left(\frac{\partial K}{\partial t}\right)dt+\left(\frac{\partial K}{\partial \varepsilon_{e}}\right)d\varepsilon_{e}$$

(3) $$\frac{\partial \varepsilon_{p}}{\partial t}=\phi {\left(\frac{\partial \varepsilon_{p}}{\partial t}\right)}_{b}+K$$

(4) $$K=-\frac{1}{C_{v}}\left(\varepsilon_{p}-C_{\lim}\varepsilon_{e}\right),K<0$$

where *C_v_* is the intrinsic creep time, representing the speed of linear creep development; *C_lim_* is the creep coefficient at infinite time.

### 2.2. Model of Corrosion Development

The corrosion products can be regarded as two different phases. Between them, the solid phase rust products accumulate at the corrosion interface. However, the liquid phase rust products have fluidity [15]. The corrosion model in this study regards the corrosion products as the secondary products of steel bar corrosion, and the corroded steel bar is expressed as the composition of the corrosion products and the original steel bar, and then its mechanical properties and deformation characteristics are considered.

#### 2.2.1. Solid Phase Corrosion Product

**Figure 2:**
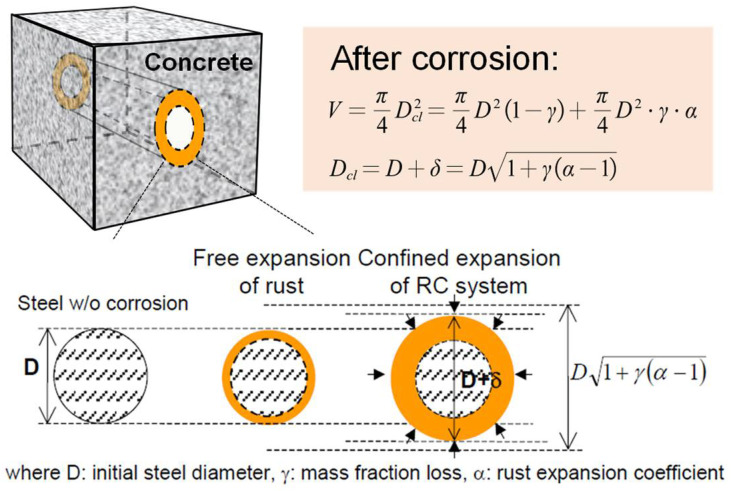
Mechanism of expansion and cracking of solid phase corrosion products [6].

Figure 2 shows the expansion of the corrosion product, which is assumed to grow uniformly around the steel bar [6]. Considering that the liquid phase rust products will flow into the pores and rust cracks around the reinforcement and relieve the stress caused by the expansion of the rust products, the volume of the solid phase rust products, namely, the rust products that always adhere to the reinforced concrete interface and generate expansion stress, is expressed as *V_s_*, as shown in Equation (5).

(5) $$V_{s}=V_{loss}-V_{l}$$

When the steel corrosion product is free to expand without restraint, the diameter of the steel bar and its composition can be expressed as

(6) $$D_{cl}=D\sqrt{1+\gamma \left(\alpha -1\right)}$$

where *D* represents the diameter of the original reinforcement; *γ* represents the volume loss rate of reinforcement, which is related to the corrosion rate. *α* represents the volume expansion rate of the corroded product relative to the corroded part of the original reinforcement.

The free expansion strain of the reinforcement rust composition can be expressed as

(7) $$\varepsilon_{s,free}=\frac{D_{cl}-D}{D}=\sqrt{1+\gamma \left(\alpha -1\right)}-1$$

where $\varepsilon_{s,free}$ is the natural corrosion expansion strain of reinforcement bar.

The average modulus of the corrosion system is defined in Equation (8), and the corresponding rust expansion stress can be obtained through free expansion strain and average modulus, as shown in Equation (9):

(8) $$E_{eq}=\frac{1+\gamma \left(\alpha -1\right)}{\left(\frac{1-\gamma }{E_{s}}\right)+\left(\frac{\gamma \alpha }{G}\right)}$$

(9) $$\sigma_{ij}=D_{ijkl}\left(E_{eq}\right)\times \left(\varepsilon_{kl}-\delta_{ij}\cdot \varepsilon_{s,free}\left(\gamma \right)\right)$$

where $E_{s}$ is the modulus of reinforcement; *G* is the modulus of solid phase corrosion product; $\sigma_{ij}^{\;}$ is the stress tensor in concrete caused by the volume expansion of solid phase corrosion products. $D_{ijkl}\left(E_{eq}\right)$ is the stiffness matrix of corroded reinforcement. $\varepsilon_{ij}$ is the actual strain of the corrosion product.

#### 2.2.2. Liquid Phase Rust Product

**Figure 3:**
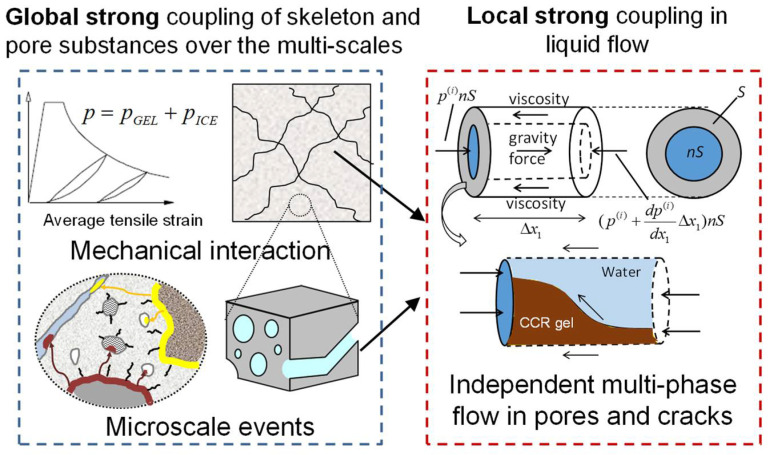
Liquid-phase corrosion product transport-cracking coupling mechanism based on pore mechanics.

The behavior of liquid phase corrosion products in concrete should be analyzed based on multiphase pore medium mechanics, as shown in Figure 3. The solid phase part (aggregate, cement paste) in the concrete is regarded as the skeleton, and the liquid phase corrosion product is regarded as the flowing pore medium, then the density ρ of the skeleton-pore system is

(10) $$\rho =\left(1-n\right)\rho_{c}+n\rho_{f}$$

where $\rho_{c}$ is the density of the skeleton (kg/m^3^), $\rho_{f}$ is the density of pore medium (kg/m^3^), and *n* is the porosity in the system.

When the concrete is not cracked, the pore pressure can be regarded as isotropic. After the concrete cracks, the pore pressure is anisotropic because the plane where the cracks are located cannot be transversely transmitted. Therefore, the stress tensor $\sigma_{ij}^{\;}$ acting on the system can be expressed as

(11) $$\sigma_{ij}=\{\begin{array}{c} \sigma_{ij}^{*}+\delta_{ij}p,\;non-crack\\ \sigma_{ij}^{*}+\delta_{ij}lp,\;crack \end{array}$$

where $\sigma_{ij}^{*}$ is the stress tensor acting on the skeleton; *p* is the pore pressure (N/mm^2^) caused by the pore medium. $\delta_{ij}^{\;}$ is a Kronecker symbol, set to 1 when *i* = *j* and 0 when *I* ≠ *j*. *l* is the unit normal vector of the crack plane.

Due to the fluidity of liquid phase corrosion products, the dynamic equilibrium equation should be used to describe the interaction between pore medium and skeleton:

(12) $$\sigma_{ij,j}+\rho g_{i}=\rho u_{i,mm}+\rho_{f}w_{i,mm}$$

(13) $$p_{,i}=\rho_{l}\left(u_{i,mm}-g_{i}\right)+\rho_{l}w_{i,mm}/n+\left(1/k_{i}\right)w_{i,m}$$

where *u_i,mm_* are the second partial derivatives of skeleton displacement with respect to time (m/s^2^); *g_i_* is the component of gravitational acceleration along vector *i* (m/s^2^); *w_i,m_* and *w_i,mm_* are the first partial derivatives (m/s) and second partial derivatives (m/s^2^) of the displacement of the pore medium relative to the skeleton with respect to time, respectively. *k_i_* is the permeability of corrosion products.

The liquid phase rust products will produce volume expansion, which will cause pore media to squeeze the pore wall (skeleton) and cause pore pressure (rust expansion stress). At the same time, the pore pressure *p* itself is constrained. According to its deformation degree, the pore pressure *p* can be obtained as

(14) $$p={\bar{K}}_{f}\left(w_{i,i}+\varepsilon_{ii}-\left(1-\beta \right)v_{cr}\right)$$

where, ${\bar{K}}_{f}$ is the volume modulus (MPa) of the skeleton-pore system. $w_{i,i}+\varepsilon_{ii}$ is the equivalent volumetric strain of pore medium. $v_{cr}$ represents the volume of solid phase corrosion products retained on the reinforced concrete surface.

In an environment saturated with pore water and rich in chlorine, the crystallization rate of rust products (i.e., the volume ratio of solid phase rust products) *β* is related to the corrosion rate of reinforcement, which can be calculated as follows [16]:

(15) $$\beta =0.75-0.1\times log_{10}\left(i_{\mathrm{corr}}\right)\leq 0.75$$

where *i*_corr_ is the corrosion rate of reinforcement (μA/cm^2^).

Due to the existence of liquid phase rust products, the rust products will be transmitted from the reinforced concrete interface to the surrounding cracks and concrete micropores [17]. From the point of view of pore mechanics, the pore pressure generated during the transport of corrosion products is only controlled by the liquid flow rate. Therefore, it is necessary to determine the transport rate *k* of the pore medium composed of liquid phase corrosion products. Relevant studies show that when concrete cracks under load, the *k* value will increase [16].

(16) $$k_{i}=k^{*}\times \left[1+{\left(\varepsilon_{jj}+\varepsilon_{kk}\right)}^{8}\right]$$

where <*i*, *j*, *k*> is the coordinate axis direction in the orthogonal coordinate system, and *j* and *k* are parallel to the crack plane; *k_i_* is the transmission rate (m/s) of the liquid phase corrosion product in the *i* direction. $\left(\varepsilon_{jj}+\varepsilon_{kk}\right)$ represents the strain of concrete along the vertical direction of the fracture plane, and $k^{*}$ can be chosen as 5 × 10^−10^ cm/s.

## 3. The Calculation Results and Analysis

### 3.1. Specimen Information

**Figure 4:**
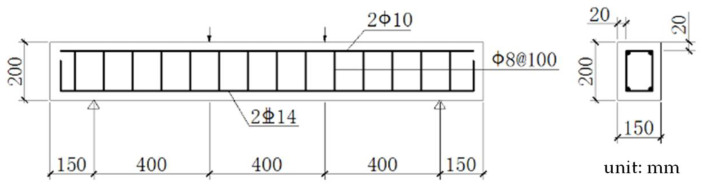
Schematic diagram of RC beam size and reinforcement.

In this paper, the reinforced concrete beam is taken as the research object, and the material parameters used in the calculation model established are the same as those of the test specimen [18]. The specific material information is shown in Table 1, and the geometric information and reinforcement of the beam are shown in Figure 4.

**Figure 5:**
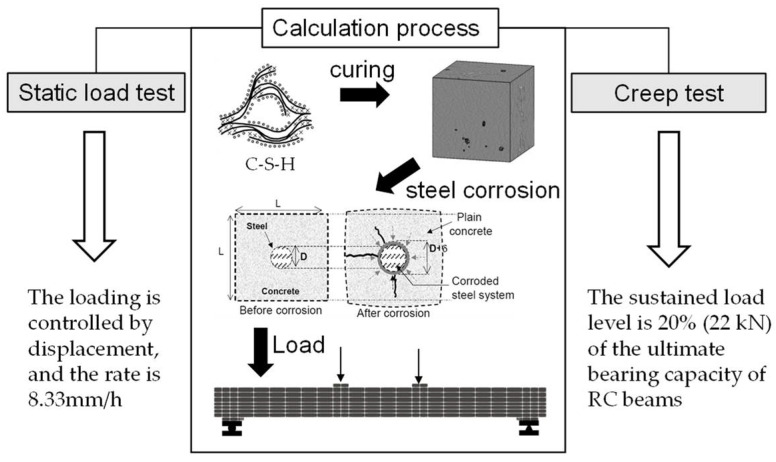
Calculation process of static load test and creep test.

In this paper, the calculation process of the static load test after the corrosion of the reinforced concrete beam is as follows: specimen curing → steel corrosion reaches preset corrosion rate → static load loading until failure. The loading form is controlled by displacement, and the rate is 8.33 mm/h. The calculation process of the creep development test for RC beams after corrosion is as follows: specimen curing → steel corrosion reaching preset corrosion rate → static load holding, and the load holding level is 20% (22 kN) of the ultimate bearing capacity of RC beams. The calculation process can be seen clearly in Figure 5. In this case, beams can be considered linear creep development.

### 3.2. Flexural Capacity and Creep Bearing Capacity of Non-Corroded Beam

**Figure 6:**
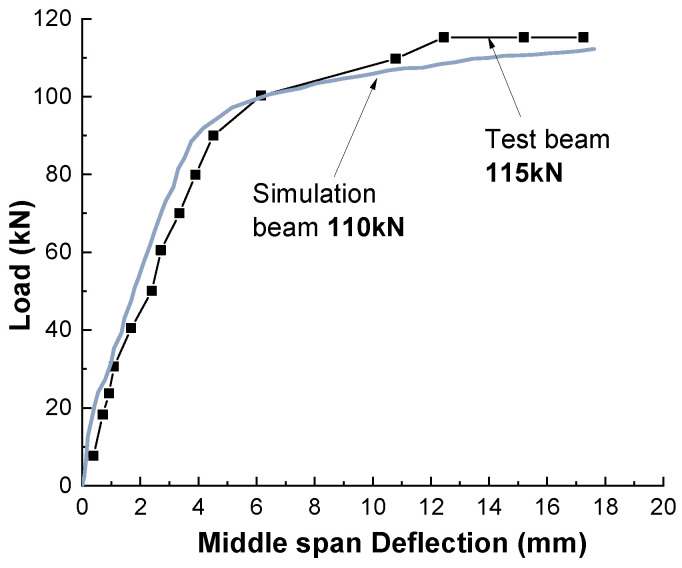
Deflection development curve of RC beam under sustained load.

**Figure 7:**
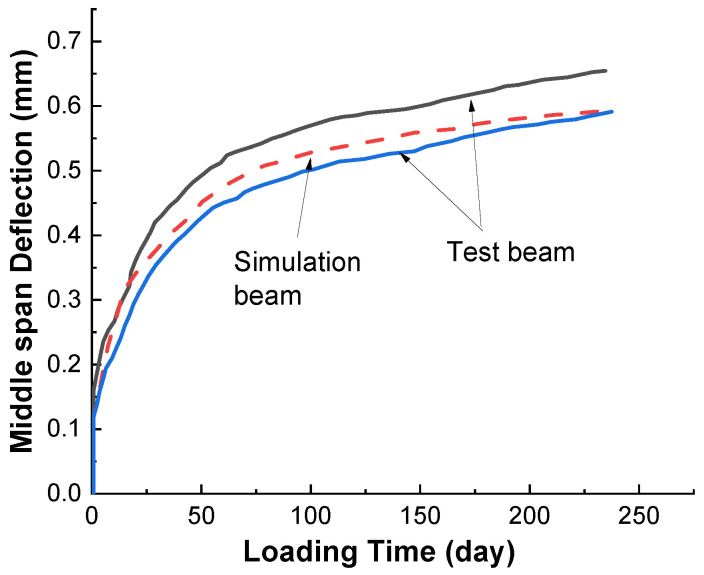
Static load test and simulation results of RC beam.

As shown in Figure 6, the failure calculation curve of beam bearing capacity is basically consistent with the test curve by calculating the failure under static load when the steel bar corrosion rate is 0%. Figure 7 shows the deflection deformation curve of the reinforced concrete beam under load state calculated by using the above rate-type creep constitutive model, which is in good agreement with the experimental results and can provide a reliable basis for subsequent corrosion and creep analysis.

### 3.3. Corrosion Cracking Pattern

**Figure 8:**
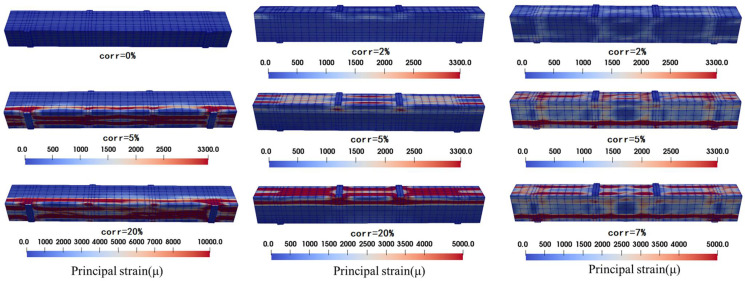
Principal strain clouds of RC beams at the end of rusting at different rusting locations.

The strain results of partial corrosion calculation are visualized as shown in Figure 8. As can be seen from the figure, the strain development in the three working conditions (bottom longitudinal bar rust, top longitudinal bar rust and all reinforcement rust) is that the principal strain of the surface concrete near the corroded reinforcement is larger than that in other areas, and obvious main cracks appear, which is consistent with previous experimental studies by other scholars [19,20]. By comparing the corrosion condition of longitudinal bars at the bottom and the corrosion condition of longitudinal bars at the top, it can be seen that the strain development at the stress position is slightly larger than that at other positions, which indicates that there is a phenomenon of local damage concentration. In addition, under the same corrosion rate, the surface strain development of the members corroded by the longitudinal bars at the bottom is slightly greater than that of the members corroded by the longitudinal bars at the top because the reinforcement diameter of the longitudinal bars at the bottom is larger (C/D is smaller) and the corrosion products are more. When all the reinforcement is corroded, the strain development of the adjacent surface of the stirrup is obvious, which is consistent with the experimental experience that the stirrup will corrode before the longitudinal reinforcement. It is proved that the stirrup slows down the corrosion process of the longitudinal reinforcement to a certain extent from the perspective of calculation.

### 3.4. Flexural Capacity of Corroded Beams

**Figure 9:**
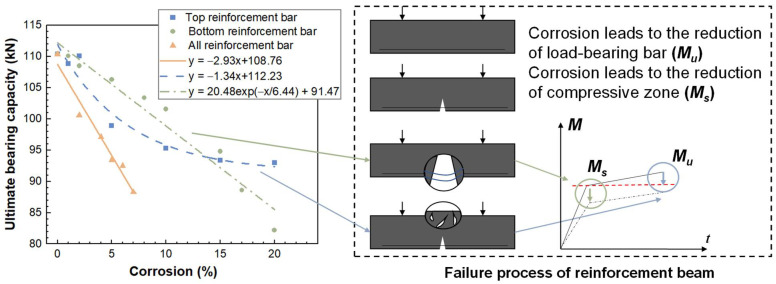
Influence of different rust patterns on the bearing capacity of RC beams.

Table 2 shows that the failure load of RC beams with different corrosion forms will decrease with the increase in corrosion rate, which means that the reinforcement corrosion in any position of the reinforced concrete beam will cause damage to the beam and reduce its bearing capacity, but the degradation law of the bearing capacity with different forms of corrosion is not the same. It can be seen from Figure 9 that the ultimate flexural capacity of the three corrosion forms exhibits different attenuation laws with the increase in corrosion rate. The ideal failure of a reinforcement concrete beam can be mainly summarized as the tensile loss of the concrete at the bottom, and then the longitudinal reinforcement at the bottom bears the tension, the compression of the concrete at the top increases, and finally, the reinforcement yields and the concrete at the top is crushed. When the longitudinal bars at the top are corroded, the compression zone of the concrete beam is damaged, and the strength of the concrete at the top mainly determines the increase in the yield load to the ultimate load of the beam (the increase in the strengthening section). With the increase in the corrosion rate, the severe fracture in the compression zone will lead to the brittle failure of the beam. The corrosion of the longitudinal reinforcement at the bottom leads to the destruction of the concrete in the tensile zone, which makes the longitudinal reinforcement work independently in advance. At the same time, the corrosion reduces the mechanical properties of the longitudinal reinforcement under stress, so the strength of the beam decreases with the increase in the corrosion rate of the longitudinal reinforcement at the bottom. The total corrosion of reinforcement can be approximated as the accumulative effect of the above two working conditions.

### 3.5. Creep Characteristics Analysis of Corroded Beams

The research on beam creep after rusting is relatively scarce, and the above contents confirm that the rate-type creep constitutive and the model considering corrosion crack and corrosion product penetration adopted by the authors have high accuracy in describing creep development and corrosion expansion. Therefore, the author intends to further study the creep properties of corroded beams and explore the influence of rust expansion and cracking on creep.

It can be seen from the foregoing that the damage caused by the corrosion of steel bars in different positions is different. The creep property degradation caused by rust corrosion is mainly due to the internal damage of concrete caused by rust expansion, so it is necessary to clarify the specific influence of crack damage in different parts on concrete creep. This section mainly explores the influence of different rust cracking modes on the creep characteristics of reinforced concrete beams.

**Figure 10:**
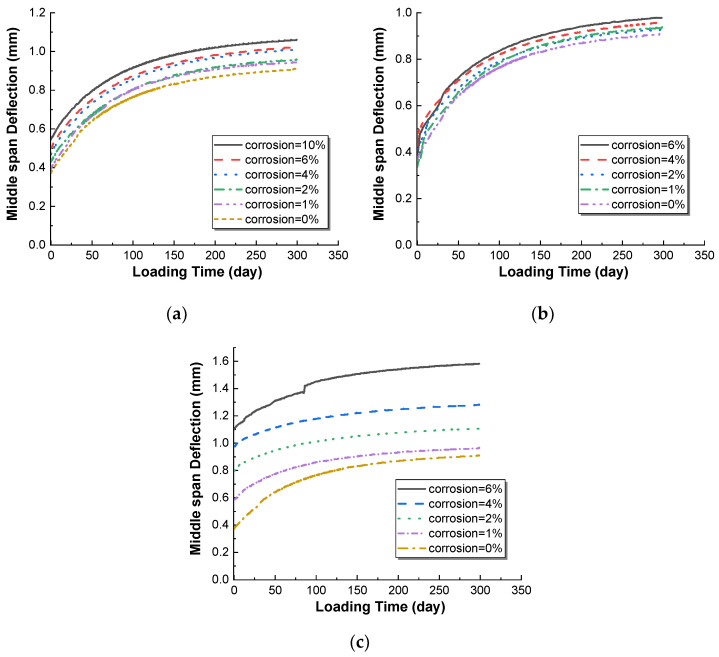
Deflection curves of RC beams with different corrosion patterns. (**a**) Corrosion of bottom bar; (**b**) Corrosion of top bar; (**c**) Corrosion of all bar.

It can be seen from Figure 10 that for the beam with only the bottom longitudinal bars corroded (Figure 10a), the deflection development curves of the steel-concrete beam under different corrosion rates are parallel, and the influence of the change in rust rate is mainly reflected in the elastic deflection value at the end of loading. It shows that the corrosion degree of the longitudinal bars at the bottom has little influence on the development of the deflection time history of the reinforced concrete beam. For the reinforced concrete beam with corroded longitudinal bars at the top (Figure 10b), the elastic deflection deformation after loading has no obvious correlation with the corrosion rate of reinforcement, and the deflection development curve is still parallel. When the rust rate is large, the deflection curve presents an uneven phenomenon at the initial stage of the sustained load, which may be because the initial rust expansion crack is further developed due to sustained load, indicating that the initial damage of concrete in the compression zone of the steel concrete beam easily develops secondary damage and undergoes abrupt deformation after the load is applied. For the fully corroded reinforced concrete beam (Figure 10c), with the continuous increase in the corrosion rate under the total corrosion condition, on the one hand, the initial elastic deflection of the component increases when the loading is completed, and on the other hand, the total deflection of the component also increases. However, the deflection development curves of each corrosion rate, as shown in the figure, are also approximately parallel. This means that the increase in the total deflection is mainly due to the increase in the initial elastic deflection value. In addition, when the corrosion rate is 6% in the total corrosion condition, the deflection development curve of the corroded reinforced concrete beam has many uneven abrupt changes, which may be caused by the relative deformation of the rust expansion crack when the rust expansion crack is more serious, which is similar to the uneven curve in the rust corrosion condition of the top longitudinal bars.

The long-term deflection of the beam is calculated by Equations (17) and (18). According to Table 3, for the beam with corroded longitudinal bars at the bottom, the larger the corrosion rate of longitudinal bars, the smaller the creep coefficient α, but the change rate decreases with the increase in the corrosion rate. For the beam with the top longitudinal reinforcement corrosion, the creep coefficient shows a trend of decrease after the first increase with the increase in corrosion rate of top longitudinal reinforcement because the top zone of the beam is the compressive zone, and a smaller corrosion rate means that the corrosion product is less. A small amount of corrosion product in the compression area of the component may not cause corrosive damage, but it will fill the original porosity and micro-cracks, and thus, the ductility of the compression zone is enhanced, and the instantaneous deformation of concrete under load decreases. This is mutually corroborated with the reduction in instantaneous deformation under load when the corrosion rate is 1% in Figure 10b. For beams with all steel bars corroded, the creep coefficient will drop sharply because of corrosion. This is because when all steel bars including stirrups are corroded, the stiffness of the members will be weakened sharply, leading to an increase in the deflection under load. The creep coefficient refers to the proportion of ductile deformation in the total deformation under continuous load. The creep coefficient under the rust condition shows a decreasing phenomenon, indicating that, in the rust environment, compared with the ductility development of beams, the stiffness of beams is weakened, and the instantaneous deformation increases sharply under load, which needs to be paid more attention to.

(17) $$\frac{\partial W_{p}}{\partial t}=-\frac{1}{\beta }\left(W_{p}-\alpha W_{e}\right)$$

(18) $$W_{p}=\alpha W_{e}\left(1-e^{-t/\beta }\right)$$

where *W_p_* is creep deflection under load; *W_e_* is instantaneous elastic deflection; *α* represents the speed of linear creep development; *β* represents the creep coefficient at infinite time

Only from the data regularity of observation, *β* had no obvious regularity. The regularity of the characterization of integration of *α* decreases with the increase in corrosion rate, and the creep coefficient is explained through the ductility in this paper, as mentioned before, but in fact, the creep coefficient is the ratio of elastic strain and plastic strain after the sustained load, and this conventional definition is used to describe traditional material creep characteristics. However, the creep coefficient used in material science is introduced in the structure in many pieces of research. The elastic strain at the moment of material loading corresponds to the instantaneous deformation at the moment of structure loading. However, for pre-damaged members, the deformation at the moment of loading is not elastic, so it is biased to use the concept of creep in this case. This is also the fundamental reason that the creep coefficient of the damaged component decreases with the increase in the initial damage. The author thinks that the quantitative index of the long-term deformation of the damaged component needs to be further improved, and the previous creep concept cannot describe the deformation characteristics of the damaged component.

**Figure 11:**
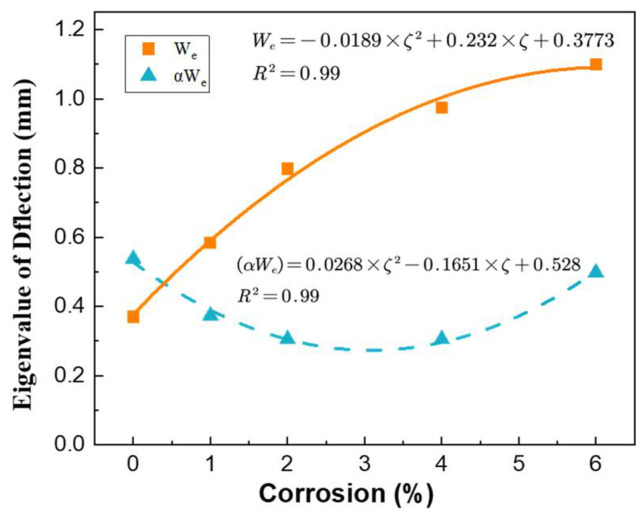
Fitting curve of *W_e_* and *αW_e_*.

**Figure 12:**
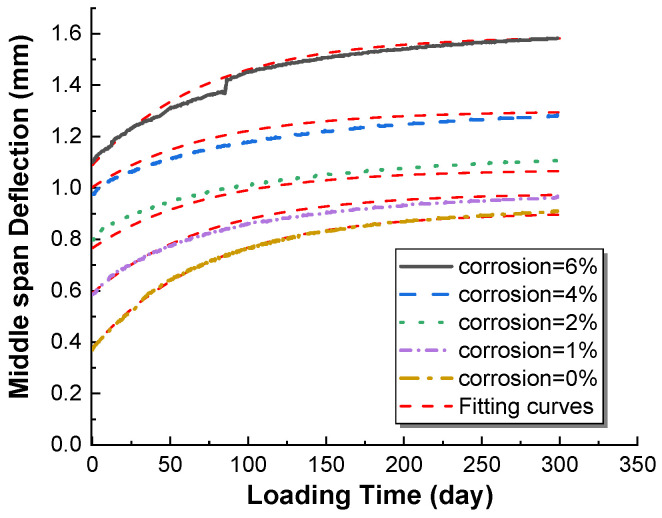
Fitting curves of equation model with different rust rates.

At present, it is more suitable for damaged members to directly study the overall deformation of the member caused by the coupling of its durability damage and sustained load, and the total deflection deformation *W_t_ = W_p_ + W_e_* can be known from the aforementioned Equation (18), as in Equation (19). In actual engineering structures, the members are basically all of the rust, and the relationship between the rust rate and deflection curve can be obtained by fitting a curve, where *ζ* is the corrosion rate. In order to facilitate the calculation of *β* take uncorroded members *β_0_*, the fitting results are shown in Equations (20) and (21). The fitted curves and the predicted effect of the deflection curve model are shown in Figure 11 and Figure 12.

(19) $$W_{t}=W_{p}+W_{e}=\left(\alpha W_{e}\right)\left(1-e^{-t/\beta_{0}}\right)+W_{e},\;\beta_{0}=74.36$$

(20) $$W_{e}=-0.0189\times \zeta^{2}+0.232\times \zeta +0.3773$$

(21) $$\left(\alpha W_{e}\right)=0.0268\times \zeta^{2}-0.1651\times \zeta +0.528$$

## 4. Conclusions

(1) In this paper, the time history effect of creep was considered based on the rate-type constitutive model, and the generation of rust expansion cracks and the penetration phenomenon of rust products were fully considered in the calculation of rust corrosion. The three-dimensional rust expansion and cracking modes caused by different rust corrosion conditions were simulated more realistically, and a logical connection was established with subsequent mechanical properties.
(2) According to the calculation and observation of the ultimate bearing capacity of the beams with different corrosion forms, the corrosion of the longitudinal bars at the top reduces the strength of the concrete in the compression zone, which leads to a decrease in the strengthening section of the fracture curve of the members and causes the brittle failure mode of the reinforced concrete beams. The corrosion of the longitudinal bars at the bottom causes the reinforcement to work in advance and reduces the mechanical properties of the tensile bars. The ultimate bearing capacity of the beam decreases gradually with the increase in the corrosion rate of the longitudinal bars at the bottom.
(3) By observing the load-bearing deformation of beams with different corrosion forms, it is found that the creep coefficient of RC beams decreases with the increase in corrosion rate. This phenomenon is mainly caused by the obvious increase in initial instantaneous deflection caused by corrosion, which does not mean that corrosion is conducive to the creep of beams. In addition, this phenomenon indicates that it is not easy to use the traditional concept of creep (deformation development/initial deformation) for the load-bearing deformation of corroded, damaged members, and it is more reasonable to use the global deflection to describe the long-term deformation of corroded, damaged members.
(4) For future research on damaged members, this paper argues that the global deflection should be used reasonably to describe the performance degradation of members, where the damage study includes rust damage, fatigue damage, etc. The coefficient of creep was originally used in materials research and migrated to structural research. The creep coefficient is only suitable for the study of sustained load at undamaged times. For the creep of damaged members, it is recommended to directly use the absolute amount of plastic deformation at the sustained load for the description.

## Figures and Tables

**Table 1:** Concrete and reinforcing steel information.

Proportions of Concrete Mix	Water Cement Ratio	Water/kg·m^−3^	Cement/kg·m^−3^	Fine Aggregate/kg·m^−3^	Coarse Aggregate/kg·m^−3^
	0.49	220	449	1125	606
Reinforcement parameters	Steel grades	Diameter/mm	Yield strength/MPa	Ultimate strength/MPa	Ductility/%
	HRB400	14	319.8	452.0	36.1
	HPB300	8/10	424.8	585.4	31.9

**Table 2:** Deterioration of ultimate bearing capacity for different rust patterns.

	Corrosion (%)	0	1	2	4	5	6	7	8	10	12	15	17	20
Bearing Capacity (kN)														
Top longitudinal reinforcement		110.4	108.9	100.1	-	98.9	-	-	-	-	-	95.4	-	93
Bottom longitudinal reinforcement		110.4	110.1	108.5	-	106.3	-	-	103.4	101.6	97.8	93.5	85.6	82.2
All reinforcement		110.4	-	100.6	97.1	93.4	92.5	88.3	-	-	-	-	-	-

**Table 3:** Variation of β and α with rust rate for beams under different rust conditions.

Corrosion Rate (%)		0	1	2	4	6	10
Top longitudinal reinforcement	*β*	74.36	64.79	69.22	69.13	59.30	-
	*α*	1.45	1.75	1.25	1.15	1.46	-
Bottom longitudinal reinforcement	*β*	74.36	73.38	84.12	77.58	78.94	77.06
	*α*	1.45	1.43	1.25	1.17	1.05	0.95
All reinforcement	*β*	74.36	73.11	79.17	87.12	90.24	-
	*α*	1.45	0.64	0.38	0.31	0.45	-

## Data Availability

Data available within the article.

## Nomenclature

*ε*	strain	$\sigma_{ij}^{\;}$	the stress tensor in concrete
*ε*_*p*_	plastic strain	$D_{ijkl}\left(E_{eq}\right)$	the stiffness matrix of corroded reinforcement
*ε_e_*	elastic strain	ρ	Density of the skeleton-pore system
*K*	damage coefficient	$\rho_{c}$	density of skeleton
*E*_0_	elastic stiffness	$\rho_{f}$	density of pore medium
*σ*	stress	*n*	the porosity in the skeleton-pore system
*t*	time	$\sigma_{ij}^{*}$	the stress tensor acting on the skeleton
*Φ*	tendency of plastic deformation	*p*	the pore pressure
*κ*	linear creep	$\delta_{ij}^{\;}$	Kronecker symbol
*C*_*v*_	intrinsic creep time	*u_i_*	skeleton displacement (i-direction)
*C*_*lim*_	creep coefficient at infinite time	*g_i_*	gravitational acceleration (i-direction)
*V*_*s*_	volume of solid phase corrosion production	*w_i_*	displacement of pore medium (i-direction)
*V*_*l*_	volume of liquid phase corrosion production	*k_i_*	permeability of corrosion products
*V*_*loss*_	volume of corrosion production	${\bar{K}}_{f}$	volume modulus of the skeleton-pore system
*D*_*cl*_	diameter of un-corroded steel	$v_{cr}$	the volume of solid phase corrosion products retained on the reinforced concrete surface
*D*	diameter of the original reinforcement	*β*	crystallization rate of rust products
*γ*	volume loss rate of reinforcement	*i*_corr_	the corrosion rate of reinforcement
*α*	volume expansion rate	*k*	transport rate
$\varepsilon_{s,free}$	the natural corrosion expansion strain of reinforcement	*W_p_*	creep deflection
$E_{s}$	modulus of reinforcement	*W*_*e*_	instantaneous elastic deflection
*G*	the modulus of solid phase corrosion product	*W_t_*	total deflection deformation
